# Bereavement, prolonged grief, and the prevention of alcohol misuse

**DOI:** 10.1111/acer.15524

**Published:** 2024-12-27

**Authors:** Jeremy W. Luk

**Affiliations:** ^1^ National Institute on Alcohol Abuse and Alcoholism Bethesda Maryland USA

In their recent article, Bottomley et al. (2024) examined the prevalence and correlates of current problematic alcohol use among 1529 adults who ever experienced the death of a significant other in their lifetime. Leveraging data from a national sample, the authors found that 30.3% of bereaved adults were screened positive for problematic alcohol use, which was assessed using the AUDIT‐C (Alcohol Use Disorders Identification Test‐Consumption) as a proxy measure. The authors suggested that this prevalence estimate was higher than rates of current binge and heavy drinking in the general US population, which underscores the possibility that the death of a significant other can be a precipitating factor for problematic alcohol use among bereaved individuals. The authors further reported that a shorter time since the death of the significant other, meeting presumptive criteria for major depressive disorder, and meeting presumptive criteria for prolonged grief disorder were independently associated with higher odds of problematic alcohol use. Besides the large sample size, the thorough consideration of bereavement‐related factors (e.g., kinship category of decedent and cause of death) and multiple psychiatric conditions (e.g., depression, posttraumatic stress disorder, and prolonged grief disorder) is of substantial merit as it helps identify features and correlates of problematic alcohol use among bereaved adults that can be used to inform prevention and intervention strategies.

The findings reported by Bottomley et al. (2024) are of relevance to clinical practice, though as an empirical paper, the authors did not provide detailed information about the diagnosis and treatment of prolonged grief disorder—a topic that may be of interest to the readers of *Alcohol: Clinical and Experimental Research*. The goal of this commentary is to elaborate on these points and is organized into three sections. First, I describe the scope of the problem and draw attention to the recent official recognition of prolonged grief disorder as a distinct diagnosis. Second, I discuss the clinical implications of findings reported by Bottomley et al. (2024) and emphasize the importance of well‐timed and evidence‐based treatment for prolonged grief disorder, noting that not all types of mental health services are equally efficacious. Third, I offer ideas to promote the integration of evidence‐based grief‐focused psychotherapy with addiction treatment and highlight the need for future research to examine bereavement and alcohol misuse in underserved populations.

## SCOPE OF THE PROBLEM AND THE OFFICIAL RECOGNITION OF PROLONGED GRIEF DISORDER

Bereavement is a near universal human experience that most individuals would encounter at some point in their lives. The loss of a loved one can be profoundly distressing and emotionally painful, and experiencing yearning for the lost loved one along with sadness and grief in the acute phase after a loss is often considered a part of normative bereavement reactions (Jordan & Litz, 2014). While these bereavement reactions typically decrease in intensity over time for most individuals, about 1 in 10 bereaved adults would experience prolonged grief that remains persistent, debilitating, and impairing to everyday functioning despite the passage of time (Lundorff et al., 2017). Among individuals who experienced bereavement following unnatural or traumatic losses (e.g., accidents, disasters, suicides, and homicides), the prevalence of prolonged grief disorder was estimated to be even higher at 49% (Djelantik et al., 2020). These findings suggest that prolonged grief in the context of bereavement is quite common and can be conceptualized as a significant life stressor that increases the risk of alcohol misuse as a maladaptive coping mechanism (Geda et al., 2024).

In the latest versions of the International Classification of Diseases (ICD‐11; 11th Revision) and the Diagnostic and Statistical Manual of Mental Disorders (DSM‐5‐TR; Fifth Edition, Text Revision), prolonged grief disorder has been officially recognized as a diagnosable condition (Szuhany et al., 2021; Zachar et al., 2023). This new condition can be defined as “a post‐loss stress syndrome in which grief after a death remains intense and preoccupying longer than expected according to social, cultural, or religious norms” (p. 1228), with the duration of clinically distressing and impairing symptoms among adults lasting for at least 6 months (ICD‐11) or 12 months (DSM‐5‐TR) (Simon & Shear, 2024). It has been proposed that the formal recognition of prolonged grief disorder as a distinct diagnosis can help identify individuals with maladaptive grief responses and help link them to appropriate resources and evidence‐based treatments (Prigerson et al., 2022). Despite these recent developments, limited prior research has examined whether rates of problematic alcohol use are higher among bereaved adults and whether prolonged grief disorder is associated with higher problematic alcohol use in this population. Addressing this gap, Bottomley et al. (2024) found that problematic alcohol use is common among bereaved adults and that prolonged grief disorder is a distinct risk factor associated with problematic alcohol use.

## IMPORTANCE OF WELL‐TIMED AND EVIDENCE‐BASED TREATMENT FOR PROLONGED GRIEF DISORDER

The identification of bereavement as a window of risk for the development of problematic alcohol use may create an opportunity for well‐timed prevention efforts following the loss of a loved one. This idea would be consistent with the finding from Bottomley et al. (2024) where individuals who experienced the loss of a significant other within the past year had the highest rate of problematic alcohol use. A major challenge in the alcohol field is the underutilization of alcohol use disorder treatment that is partly attributable to barriers such as low perceived need for care, stigma, and low perceived usefulness of available treatments (Venegas et al., 2021). The provision of treatment that integrates grief‐focused psychotherapy with alcohol prevention may help address these barriers. First, facing the loss of a significant other may increase perceived need for care in the context of an external stressor rather than a “personal failure” (Keyes et al., 2010). Second, since bereavement is a near universal experience, individuals may be more open to getting help without feeling judged or held responsible for their condition (Kilian et al., 2021). Third, offering grief‐specific support during bereavement may be seen as more relevant and useful for individuals at risk for alcohol misuse and has the potential to increase treatment utilization rates. Regarding this last point, it is notable that over half of the bereaved adults who misused alcohol in the study by Bottomley et al. (2024) utilized some form of mental health services (52.7%), a utilization rate that is substantially higher than in the general population for alcohol‐specific treatment services among those with alcohol use disorder in the past year (7.3%) (Han et al., 2021).

Bottomley et al. (2024) further provided a breakdown of the type of mental health services utilized, including individual counseling, family counseling, religion‐based mental health counseling, group counseling, medication assistance, and inpatient mental health services. While informative, this categorization of mental health services did not specify whether evidence‐based approaches were used and whether the services were provided by professionally trained clinicians or facilitators. This is an area that warrants future research. Related to the issue of categorizing treatment types, it is possible that the treatment utilization rates in Bottomley et al. (2024) appeared higher than other population‐based estimates due to the more inclusive nature of these categories (e.g., religion‐based mental health counseling may not have been included in other studies). A standardized approach to categorize mental health services and alcohol/substance use treatment modalities may help resolve these inconsistencies and facilitate more meaningful comparisons of treatment utilization rates across various studies.

The delineation of treatment modalities is important because different types of mental health services are not equally efficacious. Current evidence suggests that grief‐focused cognitive behavioral therapies (CBT) have the strongest empirical support, whereas bereavement support groups and brief contact interventions have limited support despite that they are commonly used (LaPlante et al., 2024). Core components of grief‐focused CBT typically include (1) establishing the lay of the land through psychoeducation, (2) promoting self‐regulation through reappraisal of troubling thoughts and beliefs, (3) building meaningful connections to share pain and let others help, (4) setting aspirational goals that can lead to positive emotions and a sense of purpose, (5) revisiting the world through exposure to previously avoided situations, (6) creating an acceptable account of the story of the death, and (7) reviewing positive and negative memories of the deceased (Shear, 2015). While CBT approaches to address comorbid posttraumatic stress disorder and substance use disorder are available and have been empirically evaluated (Kline et al., 2023), a more focused approach to integrate alcohol prevention with grief‐focused CBT for prolonged grief disorder has not yet been developed and tested. The need for such an integrated approach is highlighted in the analyses by Bottomley et al. (2024) which showed that presumptive prolonged grief disorder, but not presumptive posttraumatic stress disorder, was uniquely associated with higher odds of current problematic alcohol use among bereaved adults.

## INTEGRATING GRIEF‐FOCUSED CBT WITH ADDICTION TREATMENT AND FUTURE DIRECTIONS

In a recent conceptual synthesis, Luk and Thompson (2024) proposed an integrated framework to map dialectical behavior therapy (DBT) skills to clinical domains implicated in contemporary addiction research so that clinicians can effectively prioritize DBT skills to inform treatment of alcohol use disorder. These clinical domains include three addictions neuroclinical assessment domains (i.e., executive function, incentive salience, and negative emotionality) and multiple quality of life domains. As an evidence‐based “third wave” behavioral therapy, the skills training component of DBT incorporates components of motivational interviewing (e.g., Pros and Cons) and traditional cognitive behavior therapy (e.g., challenging thoughts captured as part of the Check the Facts skill and behavioral activation captured as part of the Opposite Action skill), but substantially expanded the list of skills to include Dialectical Abstinence, mindfulness techniques, and acceptance strategies. The broad range of cognitive behavioral skills available within DBT makes it an attractive option to address the complexity of comorbid prolonged grief disorder and alcohol use disorder. For instance, due to the unchangeable nature of death, DBT's Radical Acceptance skill may be well suited to help bereaved adults increase acceptance of the loss of life in a more total and complete way, thereby reducing suffering and increasing adaptive functioning. Recognizing the importance of biopsychosocial functioning and wellbeing in recovery from alcohol use disorder (Hagman et al., 2022), DBT's ABC PLEASE skill can be tailored to address quality of life issues that bereaved individuals may encounter. Specifically, *A*ccumulating positive emotions via increasing pleasurable events and valued aligned activities can increase psychological quality of life and social functioning. *B*uilding mastery can help individuals with a significant loss recover a sense of control over other domains of life. *C*oping ahead of time with difficult situations can help bereaved individuals create a plan to increase coping capacity when faced with situations that remind them of the deceased (e.g., certain places, the holiday season, or during the anniversary of the death of their loved one). Finally, the PLEASE skill encourages individuals to take care of their physical health via positive lifestyle changes (e.g., treating physical illness, balanced diet, adequate sleep, and getting exercise) to take care of their emotional health, which can be beneficial to increase physical quality of life among bereaved individuals as they reengage in the world. While clinical research is needed to establish the efficacy of this proposed approach for treating co‐occurring prolonged grief disorder and alcohol use disorder, CBT trained clinicians who work with bereaved individuals at risk for alcohol misuse may utilize clinical judgment to incorporate some of these specific skills to enhance their practice with patients when deemed appropriate.

In terms of future direction, the examination of prolonged grief disorder and alcohol misuse in underserved populations, such as older adults and individuals with alcohol‐associated liver disease, may be warranted. First, recent research has identified alcohol use and its adverse health effects among older adults as an area of increasing concern (White et al., 2023). Although bereavement can affect individuals of all ages, its negative effects on physical and mental health may be especially challenging to manage and recover from among older adults who might be more socially isolated and/or experience chronic medical conditions. Thus, the effects of bereavement and prolonged grief on the risk of alcohol misuse among older adults may be an area of increased relevance and interest. Second, a recent study found that alcohol use disorder treatment was underutilized among patients with alcohol‐associated cirrhosis, with the past 12‐month utilization rate of alcohol use disorder treatment estimated at 32% (Luk et al., 2024). Individuals with alcohol‐associated cirrhosis may experience greater medical and socioeconomic vulnerabilities that increase their risk for continued alcohol misuse. On top of these vulnerability factors, the loss of a loved one, especially if the cause of death is attributable to alcohol‐associated diseases, may lead to questions about the prognosis of their own conditions and potential alcohol misuse as a maladaptive coping mechanism to manage stress and negative emotions. Extending the important work by Bottomley et al. (2024), more research on bereavement, prolonged grief, and alcohol misuse prevention among older adults and individuals with alcohol‐associated liver disease is needed to better serve these underrepresented populations.

## CONFLICT OF INTEREST STATEMENT

The author has no conflicts of interest to disclose.

## Data Availability

N/A.

## References

[acer15524-bib-0001] Bottomley, J.S. , Williams, J.L. , Pavlacic, J.M. , Gex, K.S. & Rheingold, A.A. (2024) Bereavement and problematic alcohol use: prevalence and predictors among a national sample of bereaved adults. Alcoholism, Clinical and Experimental Research. Available from: 10.1111/acer.15496 39581744

[acer15524-bib-0002] Djelantik, A. , Smid, G.E. , Mroz, A. , Kleber, R.J. & Boelen, P.A. (2020) The prevalence of prolonged grief disorder in bereaved individuals following unnatural losses: systematic review and meta regression analysis. Journal of Affective Disorders, 265, 146–156. Available from: 10.1016/j.jad.2020.01.034 32090736

[acer15524-bib-0003] Geda, D.W. , Stangl, B.L. , Arsenault, A. , Thompson, M.F. , Schwandt, M.L. , Goldman, D. et al. (2024) Drinking motives link positive and negative life events to problematic alcohol use during the COVID‐19 pandemic: a longitudinal study. Alcohol and Alcoholism, 59(6), agae068. Available from: 10.1093/alcalc/agae068 39367531 PMC12097890

[acer15524-bib-0004] Hagman, B.T. , Falk, D. , Litten, R. & Koob, G.F. (2022) Defining recovery from alcohol use disorder: development of an NIAAA research definition. The American Journal of Psychiatry, 179(11), 807–813. Available from: 10.1176/appi.ajp.21090963 35410494

[acer15524-bib-0005] Han, B. , Jones, C.M. , Einstein, E.B. , Powell, P.A. & Compton, W.M. (2021) Use of medications for alcohol use disorder in the US: results from the 2019 National Survey on drug use and health. JAMA Psychiatry, 78(8), 922–924. Available from: 10.1001/jamapsychiatry.2021.1271 34132744 PMC8209593

[acer15524-bib-0006] Jordan, A.H. & Litz, B.T. (2014) Prolonged grief disorder: diagnostic, assessment, and treatment considerations. Professional Psychology: Research and Practice, 45(3), 180–187.

[acer15524-bib-0007] Keyes, K.M. , Hatzenbuehler, M.L. , McLaughlin, K.A. , Link, B. , Olfson, M. , Grant, B.F. et al. (2010) Stigma and treatment for alcohol disorders in the United States. American Journal of Epidemiology, 172(12), 1364–1372. Available from: 10.1093/aje/kwq304 21044992 PMC2998202

[acer15524-bib-0008] Kilian, C. , Manthey, J. , Carr, S. , Hanschmidt, F. , Rehm, J. , Speerforck, S. et al. (2021) Stigmatization of people with alcohol use disorders: an updated systematic review of population studies. Alcoholism, Clinical and Experimental Research, 45(5), 899–911. Available from: 10.1111/acer.14598 33970504

[acer15524-bib-0009] Kline, A.C. , Panza, K.E. , Lyons, R. , Kehle‐Forbes, S.M. , Hien, D.A. & Norman, S.B. (2023) Trauma‐focused treatment for comorbid post‐traumatic stress and substance use disorder. Nature Reviews Psychology, 2(1), 24–39. Available from: 10.1038/s44159-022-00129-w

[acer15524-bib-0010] LaPlante, C.D. , Hardt, M.M. , Maciejewski, P.K. & Prigerson, H.G. (2024) State of the science: psychotherapeutic interventions for prolonged grief disorder. Behavior Therapy, 55(6), 1303–1317. Available from: 10.1016/j.beth.2024.07.002 39443067 PMC11979903

[acer15524-bib-0011] Luk, J.W. , Ha, N.B. , Shui, A.M. , Snyder, H.R. , Batki, S.L. , Ostacher, M.J. et al. (2024) Demographic and clinical characteristics associated with utilization of alcohol use disorder treatment in a multicenter study of patients with alcohol‐associated cirrhosis. Alcoholism, Clinical and Experimental Research. Available from: 10.1111/acer.15500 PMC11747812 39632077

[acer15524-bib-0012] Luk, J.W. & Thompson, M.F. (2024) Mapping dialectical behavior therapy skills to clinical domains implicated in contemporary addiction research: a conceptual synthesis and promise for precision medicine. Cognitive and Behavioral Practice. Available from: 10.1016/j.cbpra.2024.07.002 PMC12334178 40857467

[acer15524-bib-0013] Lundorff, M. , Holmgren, H. , Zachariae, R. , Farver‐Vestergaard, I. & O'Connor, M. (2017) Prevalence of prolonged grief disorder in adult bereavement: a systematic review and meta‐analysis. Journal of Affective Disorders, 212, 138–149. Available from: 10.1016/j.jad.2017.01.030 28167398

[acer15524-bib-0014] Prigerson, H.G. , Shear, M.K. & Reynolds, C.F., III . (2022) Prolonged grief disorder diagnostic criteria‐helping those with maladaptive grief responses. JAMA Psychiatry, 79(4), 277–278. Available from: 10.1001/jamapsychiatry.2021.4201 35107569

[acer15524-bib-0015] Shear, M.K. (2015) Clinical practice. Complicated grief. The New England Journal of Medicine, 372(2), 153–160. Available from: 10.1056/NEJMcp1315618 25564898

[acer15524-bib-0016] Simon, N.M. & Shear, M.K. (2024) Prolonged grief disorder. The New England Journal of Medicine, 391(13), 1227–1236. Available from: 10.1056/NEJMcp2308707 39589372

[acer15524-bib-0017] Szuhany, K.L. , Malgaroli, M. , Miron, C.D. & Simon, N.M. (2021) Prolonged grief disorder: course, diagnosis, assessment, and treatment. Focus, 19(2), 161–172. Available from: 10.1176/appi.focus.20200052 34690579 PMC8475918

[acer15524-bib-0018] Venegas, A. , Donato, S. , Meredith, L.R. & Ray, L.A. (2021) Understanding low treatment seeking rates for alcohol use disorder: a narrative review of the literature and opportunities for improvement. The American Journal of Drug and Alcohol Abuse, 47(6), 664–679. Available from: 10.1080/00952990.2021.1969658 34464542 PMC9059657

[acer15524-bib-0019] White, A.M. , Orosz, A. , Powell, P.A. & Koob, G.F. (2023) Alcohol and aging—an area of increasing concern. Alcohol, 107, 19–27. Available from: 10.1016/j.alcohol.2022.07.005 35940508

[acer15524-bib-0020] Zachar, P. , First, M.B. & Kendler, K.S. (2023) Prolonged grief disorder and the DSM: a history. The Journal of Nervous and Mental Disease, 211(5), 386–392. Available from: 10.1097/nmd.0000000000001618 37040140

